# Pembrolizumab Plus Lenvatinib for Metastatic Renal Cell Carcinoma in a Patient on Hemodialysis

**DOI:** 10.1002/iju5.70011

**Published:** 2025-03-09

**Authors:** Shimpei Yamashita, Hiroki Kawabata, Yuya Iwahashi, Satoshi Muraoka, Takahito Wakamiya, Fumiyoshi Kojima, Yasuo Kohjimoto, Shin‐ichi Murata, Isao Hara

**Affiliations:** ^1^ Department of Urology Wakayama Medical University Wakayama Japan; ^2^ Department of Pathological Diagnosis Wakayama Medical University Wakayama Japan

**Keywords:** hemodialysis, lenvatinib, pembrolizumab, renal cell carcinoma, safety

## Abstract

**Introduction:**

There is no clear evidence of the safety and efficacy of pembrolizumab plus lenvatinib combination therapy in patients on hemodialysis. This is the first report of a patient on hemodialysis to receive this combination therapy for metastatic renal cell carcinoma.

**Case Presentation:**

A 60‐year‐old man on hemodialysis had cT3bN1M0 unclassified renal cell carcinoma. He received the combination therapy of pembrolizumab plus lenvatinib. Although the dose of lenvatinib was halved to 10 mg/day due to Grade 3 thrombocytopenia, 6 months later, the volumes of the primary lesion and lymph node metastasis were remarkably reduced.

**Conclusion:**

Our case report suggests the feasibility of the combination therapy of pembrolizumab plus lenvatinib for patients with metastatic renal cell carcinoma who are undergoing hemodialysis.

**Summary**

- A 60‐year‐old man on hemodialysis with metastatic unclassified renal cell carcinoma received the combination therapy of pembrolizumab plus lenvatinib.
- The volumes of the primary lesion and lymph node metastasis were remarkably reduced without unexpected severe adverse events.
- The present case suggests the feasibility of this combination therapy for patients with metastatic renal cell carcinoma who are undergoing hemodialysis.

**Abbreviations**
ICI: immune checkpoint inhibitor
PD‐1: programmed cell death 1
RCC: renal cell carcinoma
TKI: tyrosine kinase inhibitor

## 1 Introduction

Combination therapies using immune checkpoint inhibitors (ICIs) now play a key role as first‐line therapy for metastatic or advanced renal cell carcinoma (RCC). Among them, the combination of pembrolizumab, an anti‐programmed cell death 1 (PD‐1) monoclonal antibody, plus lenvatinib, an antiangiogenic agent, was associated with better efficacy than sunitinib alone as first‐line treatment in a Phase 3 trial [1]. This combination therapy was also shown in a Phase 2 trial to have a promising antitumor effect in previously untreated patients with non–clear cell RCC [2]. However, to the best of our knowledge, there is no clear evidence of the safety and efficacy of pembrolizumab plus lenvatinib combination therapy in patients on hemodialysis. Here, we report the case that suggests its feasibility for patients with metastatic RCC who are undergoing hemodialysis.

## 2 Case Report

**FIGURE 1:**
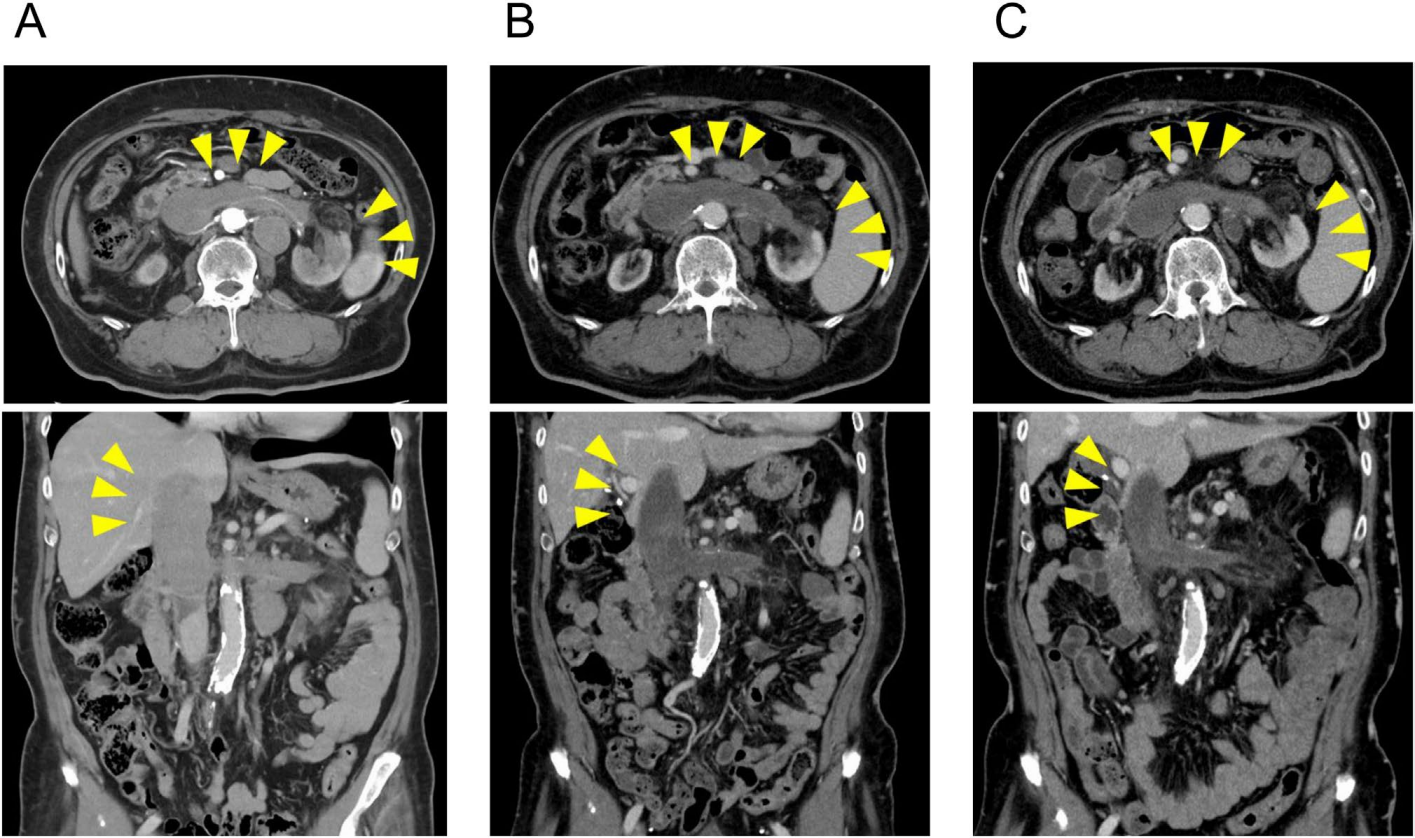
Dynamic computed tomography images of the primary kidney lesion: (A) before treatment with pembrolizumab plus lenvatinib, (B) 2 months after initiation of the combination therapy, and (C) 6 months after initiation of the combination therapy.

**FIGURE 2:**
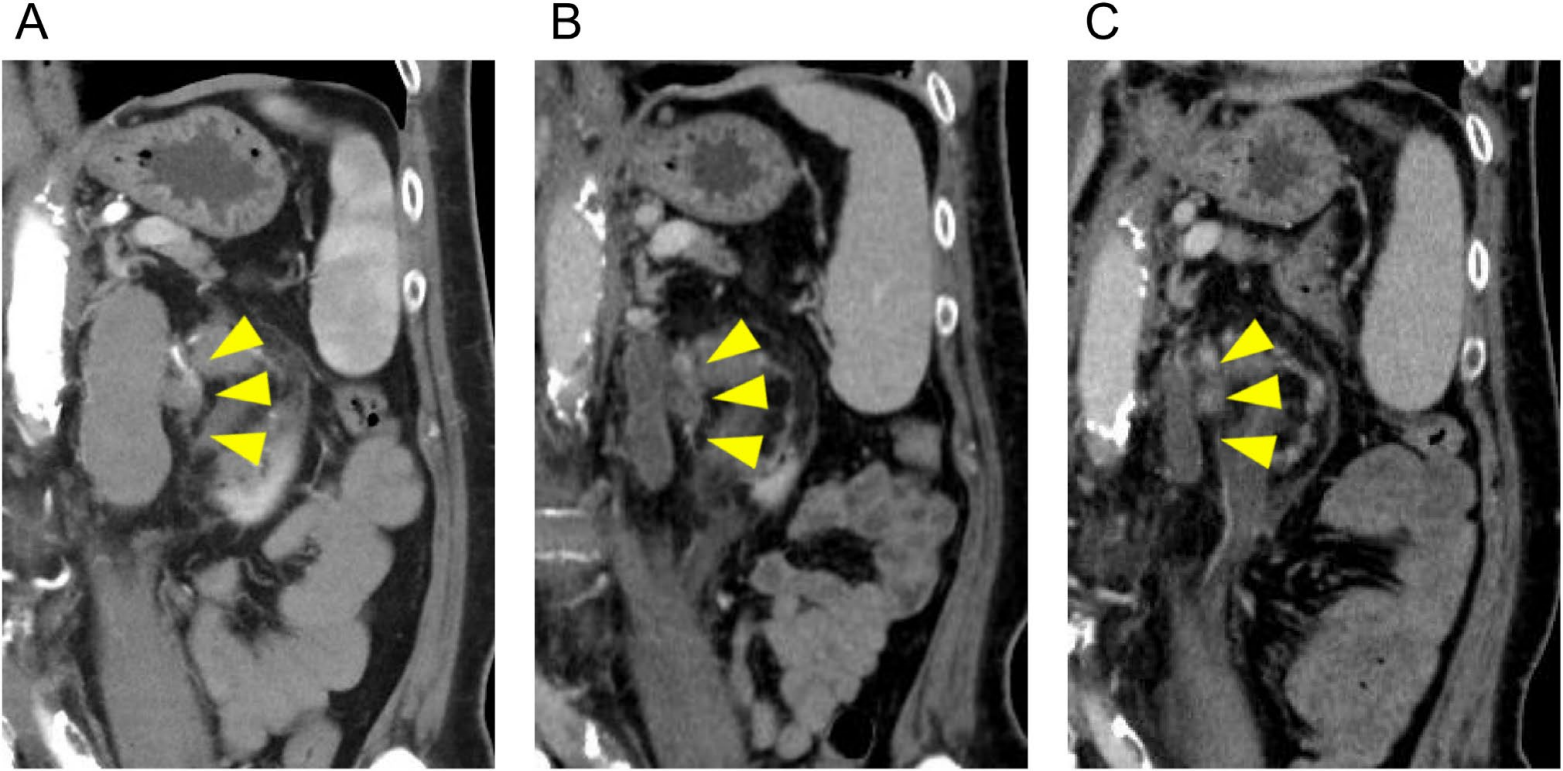
Dynamic computed tomography images of renal hilar lymph node: (A) before treatment with pembrolizumab plus lenvatinib, (B) 2 months after initiation of the combination therapy, and (C) 6 months after initiation of the combination therapy.

**FIGURE 3:**
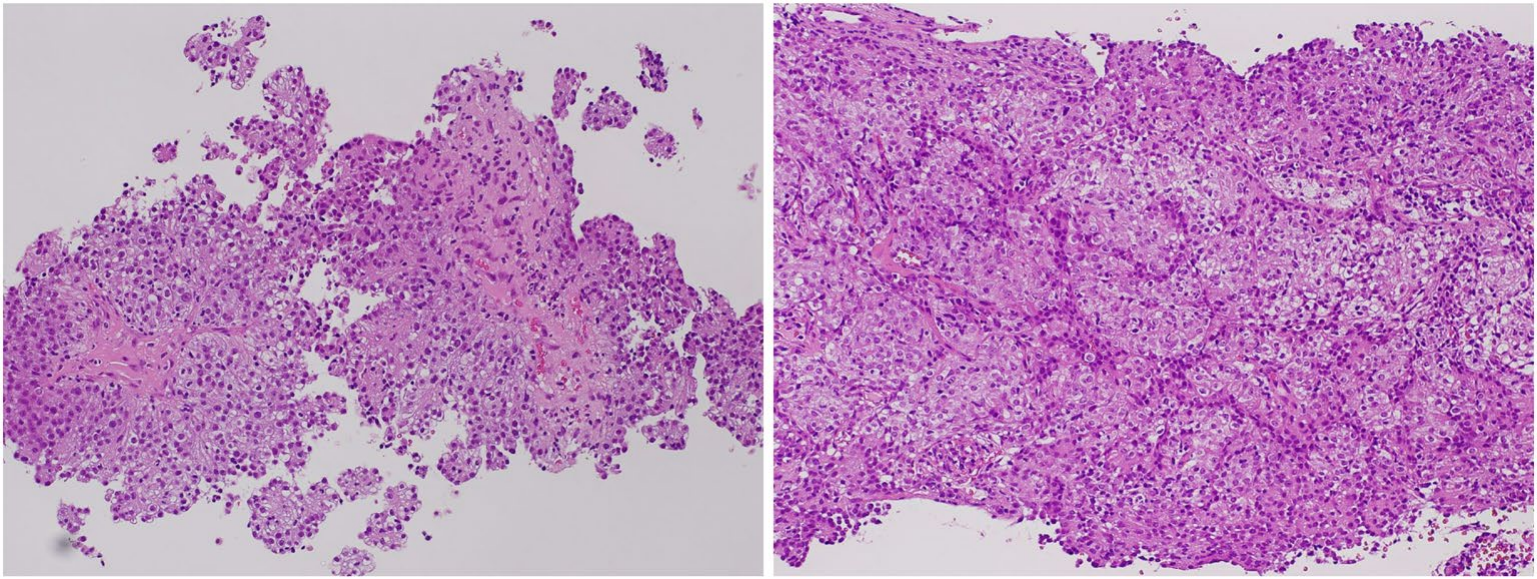
Hematoxylin‐ and eosin‐stained tissue section of biopsy specimen with low magnification (×100).

A 60‐year‐old man with a body mass index of 26.6 was referred to our hospital for a detailed examination of his left renal tumor. Dynamic computed tomography had been performed elsewhere to examine the asymptomatic elevation of serum C‐reactive protein (7.4 mg/dL), and it revealed a left renal tumor extending into the inferior vena cava (Figure 1A) and hilar lymph node enlargement (Figure 2A). He was started on hemodialysis due to the progression of diabetic nephropathy 5 years before the current presentation. A computed tomography–guided percutaneous lymph node biopsy was performed because the primary renal lesion was small and located on the ventral side, and it revealed metastasis of unclassified RCC (Figure 3). He was diagnosed with cT3bN1M0 RCC and categorized as intermediate risk (decreased hemoglobin level and time from initial diagnosis to initiation of systemic therapy < 1 year) according to the International Metastatic Renal Cell Carcinoma Database Consortium classification. The combination therapy of pembrolizumab (200 mg/3 weeks) plus lenvatinib (20 mg/day) was initiated as first‐line therapy for metastatic RCC. Due to Grade 2 thrombocytopenia, the dose of lenvatinib was reduced from 20 to 14 mg/day 15 weeks after treatment initiation. However, Grade 3 thrombocytopenia developed 6 weeks later, necessitating a 1‐week interruption of lenvatinib. After recovery of thrombocytopenia, the dose of lenvatinib was further reduced to 10 mg/day. No other treatment‐related adverse events were observed during the treatment. Two months after initiating the combination therapy, a significant reduction in the volume of the primary lesion and lymph node metastases was observed (Figures 1B and 2B). Six months after initiation of the treatment, the tumors had further decreased in size, which was judged to be a partial response (Figures 1C and 2C). In addition, serum C‐reactive protein was also normalized (0.02 mg/dL) 6 months after initiating the treatment. Now, approximately 1 year after the initiation of treatment, the patient is able to continue the combination therapy without any additional adverse events.

## 3 Discussion

Our patient, who is on hemodialysis, benefited from receiving pembrolizumab and lenvatinib combination therapy for metastatic unclassified RCC. To the best of our knowledge, this is the first case report to show the clinical outcomes of this combination therapy in a patient on hemodialysis.

The CLEAR trial, a multicenter, open‐label, randomized, Phase 3 trial, showed that pembrolizumab plus lenvatinib was associated with a significantly higher objective response rate, longer progression‐free survival, and longer overall survival than sunitinib alone in patients with advanced or metastatic clear cell RCC who had received no previous systemic therapy [1]. The KEYNOTE‐B61 trial, a multicenter, single‐arm, Phase 2 trial, demonstrated that this combination therapy had a promising antitumor effect in previously untreated patients with non–clear cell RCC [2]. However, adequate renal function was an inclusion criterion in these trials, so patients on hemodialysis were excluded from them. The safety and efficacy of pembrolizumab plus lenvatinib combination therapy in patients on hemodialysis have therefore been unclear, and there is a lack of reports. However, the efficacy and safety of pembrolizumab alone and other tyrosine kinase inhibitors (TKIs) in patients undergoing hemodialysis have already been reported, as summarized below.

The efficacy and safety of pembrolizumab for patients on hemodialysis with non–small cell lung cancer were reportedly comparable with those with normal renal function [3]. Similarly, there is a report on the efficacy and safety of pembrolizumab in a patient undergoing hemodialysis who had melanoma [4]. The antitumor effect of pembrolizumab‐bound lymphocytes is considered to be undiminished in patients undergoing hemodialysis because a humanized immunoglobulin G4 monoclonal antibody against human PD‐1 cannot pass through the glomerular membrane of the kidney and is not removed by dialysis because of its high molecular weight [4]. In addition, the clinical outcomes of other ICIs in patients on hemodialysis with metastatic RCC have been reported in several studies. For example, one reported the efficacy and safety of nivolumab, an anti‐PD‐1 monoclonal antibody, in eight patients with metastatic RCC who were undergoing hemodialysis. Treatment with nivolumab was concluded to be feasible with good efficacy, and there was no unexpected toxicity in patients undergoing hemodialysis [5]. Another paper reported treatment with nivolumab plus ipilimumab, an anti‐cytotoxic T‐lymphocyte antigen‐4 antibody, for a patient on hemodialysis with advanced clear cell RCC; it showed the efficacy and safety of this combined ICI therapy [6].

The clinical outcomes of TKIs other than lenvatinib in patients with metastatic RCC and undergoing hemodialysis have also been previously reported. The efficacy and safety of sunitinib and sorafenib for hemodialyzed patients with RCC were summarized, and TKI treatment for patients undergoing hemodialysis was said to be safe and effective [7]. The efficacy and safety of axitinib in patients on hemodialysis with metastatic RCC have also been supported by several other reports [8, 9]. Meanwhile, lenvatinib has different characteristics from these TKIs. Lenvatinib is a multikinase inhibitor that inhibits not only vascular endothelial growth factor receptors 1–3 but also fibroblast growth factor receptors 1–4 [10]. Pharmacodynamic and pharmacokinetic properties of lenvatinib are also different from those of other TKIs. Despite this background, there are no previous reports on the efficacy and safety of lenvatinib in patients with metastatic RCC who are undergoing hemodialysis.

In the present case, lenvatinib was started at the usual dose of 20 mg/day, and although the dose was halved to 10 mg/day due to thrombocytopenia, a good antitumor effect was observed without other notable adverse events. Unfortunately, pharmacokinetic and pharmacodynamic analyses of lenvatinib were not performed, so further accumulation of case reports about the clinical outcomes of lenvatinib in patients undergoing hemodialysis is required.

Of course, patients undergoing hemodialysis require specific considerations, including their susceptibility to infection and cardiovascular fragility, as well as their potential for atherosclerosis syndrome, malnutrition, and unexpected symptoms requiring emergency treatment [11]. If the ICI plus TKI combination therapy is used for patients undergoing hemodialysis, close follow‐up and fine dose adjustment of TKI are required, as well as drug withdrawal when necessary.

## 4 Conclusion

Our case report suggests the feasibility of the combination therapy of pembrolizumab plus lenvatinib for patients with metastatic RCC who are undergoing hemodialysis.

## Consent

Written informed consent to publication of this case report, including clinical details and clinical images, was obtained from the patient. A copy of the written consent form is available for review upon reasonable request.

## Conflicts of Interest

The authors declare no conflicts of interest.
